# Improving the diagnosis of cassava mosaic begomoviruses using Oxford Nanopore Technology sequencing

**DOI:** 10.1038/s41598-025-25233-8

**Published:** 2025-11-21

**Authors:** Mariam Combala, Ezechiel B. Tibiri, Justin S. Pita, Angela O. Eni, Seydou Sawadogo, Pakyendou E. Name, Saïdou Zongo, Poupouanou Kouhoumouri, Adama Sagnon, Oghenevwairhe P. Efekemo, Olabode Onile-ere, Allen Oppong, Cyrielle Ndougonna, Fidèle Tiendrébéogo

**Affiliations:** 1 Central and West African Virus Epidemiology (WAVE) for Food Security Program, Pôle Scientifique et d’Innovation, Université Félix Houphouët-Boigny, Bingerville, Côte d’Ivoire; 2 UFR Biosciences, Université Félix Houphouët-Boigny, Abidjan, Côte d’Ivoire; 3 Laboratoire de Virologie et de Biotechnologies Végétales (LVBV), Institut de L’Environnement et de Recherches Agricoles (INERA), 04 BP 8645 Ouagadougou, Burkina Faso; 4 Central and West African Virus Epidemiology Program, Covenant University Hub (WAVE-CU), Km. 10 Idiroko Road, Canaan Land, Ota, Ogun State Nigeria; 5 CSIR-Crops Research Institute, P.O. Box 3785, Kumasi-GhanaKumasi, Ghana

**Keywords:** Cassava mosaic disease, Begomovirus, Oxford Nanopore Technology sequencing, Palindromic motifs, Specific primers, Biological techniques, Microbiology, Molecular biology

## Abstract

Cassava mosaic disease (CMD), caused by begomoviruses such as African cassava mosaic virus (ACMV) and East African cassava mosaic virus (EACMV), poses a threat to food security in sub-Saharan Africa. Conventional PCR assays often fail to detect viral strains in symptomatic plants due to high genetic variability and recombination. In this study, we used Oxford Nanopore Technology (ONT) sequencing on 12 cassava leaf samples that had previously tested negative by PCR. We compared two strategies: direct sequencing of total plant DNA and sequencing after rolling circle amplification (RCA-MinION). Across the samples, we obtained 7,800–36,000 reads, of which 1,327–11,749 were viral reads after host filtering. While direct sequencing of total DNA detected CMD-associated reads without yielding full genomes, RCA-MinION enabled de novo assembly of complete ACMV and EACMV genomes (two to 14 contigs, N50 up to 22.2 kb). This revealed high genetic diversity, mixed infections and recombination. Building on these genomic datasets, we performed computational analyses to identify conserved genomic regions and palindromic motifs, which guided the rational design of new primers. These primers, which target the AV1, AC2, BV1 and BC1 regions, were validated in silico and by PCR. They achieved detection rates of up to 98% across diverse isolates and successfully amplified viral DNA in samples that had previously been undetected by standard primers. Palindromic motif analysis further reduced the risk of secondary structures, ensuring efficient primer binding. Sanger sequencing of the PCR products confirmed the specificity and robustness of the assays. Our findings suggest that ONT combined with RCA is a powerful tool for CMD diagnostics and surveillance, improving detection and providing the genomic insights that are critical for disease management and food security in West Africa.

**Supplementary Information:**

The online version contains supplementary material available at 10.1038/s41598-025-25233-8.

## Introduction

Cassava (*Manihot esculenta* Crantz) is a staple crop that sustains the livelihoods of more than 800 million people in sub-Saharan Africa^1^⁠. Its resilience to drought and adaptability to marginal soils make it a critical resource for food security in the region. In addition, cassava’s role in supporting rural economies and ensuring food stability underscores its importance as a strategic crop for poverty reduction and sustainable development in Africa. Despite its importance, cassava productivity is severely undermined by biotic stresses, particularly viral diseases such as cassava mosaic disease (CMD).

CMD is caused by cassava mosaic begomoviruses (CMBs), including African cassava mosaic virus (ACMV) and East African cassava mosaic virus (EACMV) and their variants. These viruses are mainly transmitted by the whitefly *Bemisia tabaci* complex and through the use of infected cuttings during propagation. The high rate of virus transmission, coupled with the vegetative nature of cassava cultivation, facilitates the rapid spread of CMD, leading to significant yield losses and exacerbating food insecurity in regions heavily dependent on cassava^2,3^⁠.

Effective disease management is further complicated by the remarkable genetic diversity and high recombination rates of CMBs. These factors contribute to the emergence of novel, highly virulent strains that often evade detection by conventional diagnostic tools^4^⁠. While PCR-based methods are widely used as the gold standard for virus identification, they have limitations in sensitivity and specificity, particularly when dealing with mixed infections or recombinant variants^5,6^⁠. Such diagnostic shortcomings hinder timely intervention and complicate breeding programmes’ efforts to develop resistant cassava varieties. There is therefore an urgent need for advanced, field-adaptable diagnostic technologies that can provide rapid and accurate virus detection to mitigate the spread of CMD.

Oxford Nanopore Technology (ONT) has emerged as a transformative approach to viral diagnostics, offering real-time sequencing capabilities, portability, and the ability to identify diverse viral populations with high resolution^6^⁠. Unlike conventional methods, ONT enables comprehensive genomic analysis in resource-limited settings, providing actionable insights into viral diversity and evolution. By facilitating in-depth characterisation of viral strains, ONT enhances our ability to monitor epidemiological trends, detect recombinant variants, and more effectively guide disease management strategies.

This study demonstrates the effectiveness of ONT in overcoming the challenges posed by viral diversity and recombination, enabling the comprehensive characterisation of CMBs strains present in symptomatic cassava samples that previously evaded detection. By integrating molecular and computational approaches, we demonstrate the utility of ONT in addressing gaps in existing diagnostic frameworks. The results presented here highlight the potential of ONT-based diagnostics to revolutionise virus surveillance and management strategies in cassava-growing regions of Africa. This advance contributes not only to sustainable agriculture but also to broader efforts to strengthen food security and resilience against plant diseases in sub-Saharan Africa.

## Results

### Nanopore sequencing results

Oxford Nanopore sequencing was performed on 12 cassava leaf samples selected from 50 symptomatic plants that initially tested negative by PCR. Two approaches were compared: direct sequencing of total plant DNA and sequencing following rolling circle amplification (RCA-MinION). Across individual samples, the raw output ranged from ~ 7,800 to 36,000 reads, with an average of ~ 20,500 reads per sample. After removing host sequences, the number of viral-associated reads varied widely (1,327–11,749 reads; mean ~ 3,475), but was sufficient to allow downstream analyses (Table 1). Both approaches revealed the presence of cassava mosaic begomoviruses (CMBs), including African cassava mosaic virus (ACMV) and East African cassava mosaic virus (EACMV). Metagenomic profiling highlighted the genomic diversity of these viruses and showed that several samples carried multiple strains, suggesting ongoing mutations and frequent mixed infections. Such complexity has direct implications for both diagnosis and disease management. The RCA-MinION strategy proved particularly effective for genome recovery. De novo assemblies produced 2–14 contigs per sample, with total contig lengths ranging from ~ 10 kb to ~ 41 kb. Contig N50 values spanned 3.0–22.2 kb, sufficient to reconstruct nearly complete viral genomes and to perform meaningful phylogenetic analyses (supplemental data 1). By contrast, direct sequencing of total plant DNA, while able to detect reads taxonomically assigned to CMD-associated viruses, did not generate assemblies of sufficient length or quality to reconstruct full genomes. This underscores the added value of RCA-MinION, which enriches viral templates and enables high-quality assemblies suitable for evolutionary studies and epidemiological surveillance. Phylogenetic inference performed on RCA-assembled genomes provided further insights into the evolutionary dynamics of cassava mosaic begomoviruses. For ACMV/EACMV DNA-A (63 full-length genomes, 4,096 bp), ModelFinder selected GTR + F + I + R4 as the best-fit model (log-likelihood − 25,187.78 ± 489.20). The consensus tree contained 60 internal nodes, of which 8 (13.3%) reached strong support (UFBoot ≥ 95%). The choice of a free-rate model with an invariant proportion reflects substantial heterogeneity across sites, with conserved regions (AC1/AV1) coexisting with rapidly evolving domains, consistent with the diversification of ACMV/EACMV lineages. For ACMV DNA-B (28 full-length genomes, 2,934 bp), the selected model was GTR + F + I + G4 (log-likelihood − 11,428.77 ± 312.85). The consensus topology contained 25 internal nodes, 7 of which (28%) were strongly supported (UFBoot ≥ 95%). The gamma-rate model with a non-zero invariant fraction captured the coexistence of conserved and variable sites in BV1/BC1. Compared with DNA-A, DNA-B displayed a more diffuse phylogenetic signal, likely reflecting the bipartite genome structure and the influence of recombination. These results emphasize the need for recombination-aware methods (GARD) and gene-wise trees to refine poorly supported relationships (Supplemental data S1).

**Table 1:** Summary of read processing and de novo assembly statistics for cassava leaf samples sequenced using Oxford Nanopore technology.

Accession	Bioproject accession	Biosample accession	Reads raw	reads_host mapped	reads_host unmapped	Read N50_bp	Read max_bp	ctg_count	Total bp_contigs	ctg_total_bp	ctg_N50_bp
SRR35173598	PRJNA1312059	SAMN50848258	24,876	23,282	1,594	5,405	5,561	4	17,929	5,561	5,405
SRR35173597	PRJNA1312059	SAMN50848259	19,604	7,855	11,749	3,352	5,650	10	21,899	5,650	3,352
SRR35173596	PRJNA1312059	SAMN50848260	32,167	30,188	1,979	3,012	4,680	6	16,065	4,680	3,012
SRR35173595	PRJNA1312059	SAMN50848261	36,085	33,674	2,411	3,585	7,510	14	41,710	7,510	3,585
SRR35173594	PRJNA1312059	SAMN50848262	21,456	20,128	1,328	3,610	3,713	3	10,134	3,713	3,610
SRR35173593	PRJNA1312059	SAMN50848263	11,660	9,800	1,860	5,961	8,172	6	27,386	8,172	5,961
SRR35173592	PRJNA1312059	SAMN50848264	28,450	26,706	1,744	8,223	8,343	6	29,343	8,343	8,223
SRR35173591	PRJNA1312059	SAMN50848265	10,442	4,894	5,548	22,248	22,248	2	41,323	22,248	22,248
SRR35173590	PRJNA1312059	SAMN50848266	7,838	3,673	4,165	13,905	13,905	5	20,292	13,905	13,905
SRR35173589	PRJNA1312059	SAMN50848267	12,533	10,160	2,373	10,904	10,904	2	16,466	10,904	10,904

The table reports the number of raw reads generated per barcode, the number of reads mapped to the cassava or human reference genomes, and the number of non-host/non-human reads retained for viral analysis. Read-level statistics include the number of final reads, N50 length and maximum read length. Assembly-level statistics include the number of contigs produced, total contig length, maximum contig length and contig N50. These metrics provide an overview of sequencing depth, host read removal efficiency and assembly quality for downstream viral genome reconstruction and taxonomic assignment. (http://www.ncbi.nlm.nih.gov/bioproject/1312059).

**Fig. 1:**
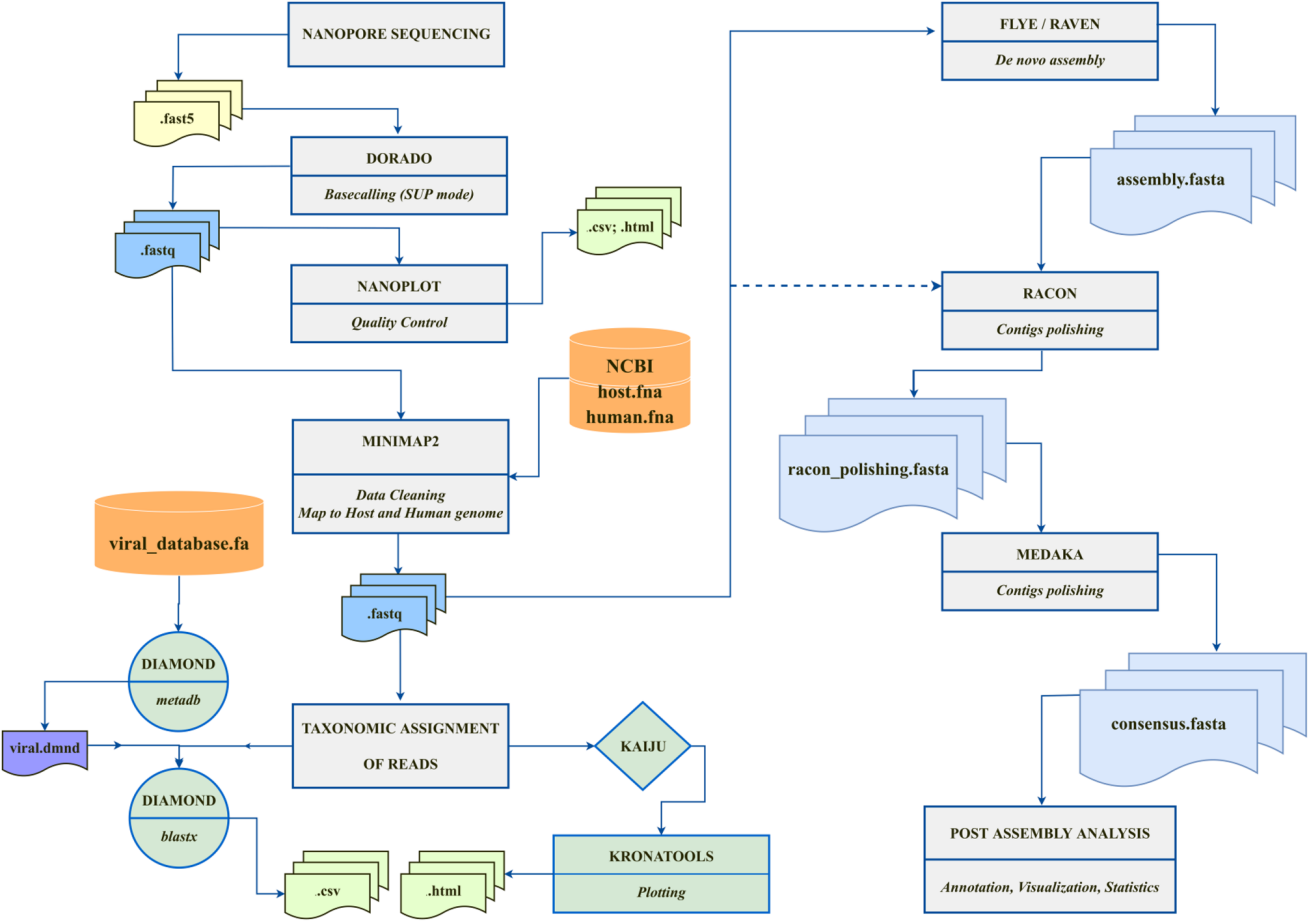
Bioinformatics workflow for Nanopore sequencing data analysis. Flowchart illustrating the bioinformatics pipeline for processing Nanopore sequencing data, from raw signal to processing to De novo assembly. The workflow includes key steps such as basecalling, quality control, adapter trimming, sequence alignment, taxonomic classification and de novo assembly. Each stage is designed to ensure the accurate and efficient analysis of sequencing data, enabling downstream applications in genomic and metagenomic studies.

Taken together, these findings highlight the potential of ONT sequencing, especially when combined with RCA enrichment, to uncover hidden viral diversity, detect mixed infections, and provide robust evolutionary insights. Information generated from ONT sequencing, together with curated GenBank datasets, also formed the basis for in-depth sequence analyses used to identify conserved genomic regions and guide the design of new diagnostic primers (Fig. 1).

### Primer design and validation

**Fig. 2:**
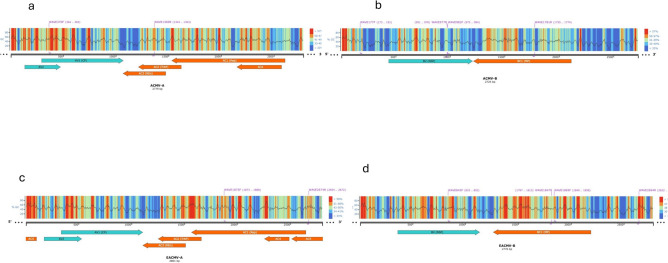
Genomic architecture of cassava mosaic begomoviruses and primer binding regions. Visual representation of the genomic features of cassava mosaic begomoviruses (**a**: ACMV-A, **b**: ACMV-B, **c**: EACMV-A, **d**: EACMV-B) with annotated primer binding sites. The schematic highlights the %GC content distribution, key open reading frames (ORFs), and the position of primers designed for diagnostic applications. Genomic elements such as replication-associated protein (Rep), coat protein (CP), movement protein (MP), and transcriptional activator protein (TrAP) are depicted for clarity in primer localisation and genome organisation**.**

New primer pairs were designed to improve diagnostic performance (Table 2). For ACMV DNA-A, primer pairs WAVE-AA508F/WAVE-AA1307R and WAVE-AA370F/WAVE-AA1369R targeted the AV1 and AC2 genes, respectively (Fig. 2), with detection rates ranging from 60 to 96% across samples (Table 2). For ACMV DNA-B, primers WAVE-AB177F/WAVE-AB977R and WAVE-AB982F/WAVE-AB1781R targeted the BV1 and BC1 genes, respectively (Fig. 2), yielding amplification rates between 48 and 98%. EACMV DNA-A primers WAVE-EA1875F/WAVE-EA2674R achieved detection rates between 40.63% and 95.45%. Meanwhile, EACMV DNA-B primers WAVE-EB845F/WAVE-EB1847R and WAVE-EB1869F/WAVE-EB2694R showed efficiencies between 47 and 95% (Table 2).

**Table 2:** List of new primer pairs designed for detection of cassava mosaic begomoviruses (CMBs).

Primer name	Primer sequence	Size (bp)	Target region
WAVE-AA508F	AAGGCCCATGTAAGGTCCAG	800	ACMV
WAVE-AA1307R	GAAGGAGCTGGGGATTCACA		AV1/AC3
WAVE-AA370F	ACAGCCCATACAGGAACCGT	1000	ACMV
WAVE-AA1369R	CGACCATTCCTGCTGAACCA		AV1/AC3
WAVE-AB177F	GATCTGCGGGCCTATCGAAT	800	ACMV
WAVE-AB977R	TTCACGCTGTGCAATACCCT		BV1
WAVE-AB982F	TTCGTGTCATCTGCAGGAGA	800	ACMV
WAVE-AB1781R	GTACCATGGCAGCTGCTGTA		BV1/BC1
WAVE-EA1875F	TGTACCAGGCGTCGTTTGAA	800	EACMV
WAVE-EA2674R	TGTCCCCCGATCCAAAACG		AC1
WAVE-EB1869F	TTCCAAGGGGAGGGTTCTGA	800	EACMV
WAVE-EB2694R	TGCTCTCGCCTCTCTCTTCT		BC1
WAVE-EB845F	CGTGTATGGCATGCCTAGGT	1000	EACMV
WAVE-EB1847R	CTCCAACGTCATAGAAGGCGT		BV1/BC1

This table provides detailed information about the primer pairs designed for the amplification of conserved genomic regions in African cassava mosaic virus (ACMV) and East African cassava mosaic virus (EACMV). Primer pairs target specific regions, including AV1, AC3, BV1, BC1, and AC1 genes, with amplicon sizes ranging from 800 to 1000 bp. These primers were optimised to improve diagnostic sensitivity and specificity, particularly for identifying recombinant variants and mixed infections that may evade conventional PCR methods. The table lists the primer names, sequences, amplicon sizes, and the corresponding target regions.

Extensive testing of these primers demonstrated their ability to amplify highly conserved regions while minimising non-specific amplification. The primers successfully detected viral DNA in samples previously considered recalcitrant, thereby improving diagnostic reliability. In silico PCR simulations further confirmed the specificity of the primers, highlighting their potential for large applications and epidemiological studies.

### Palindromic motif analysis

**Fig. 3:**
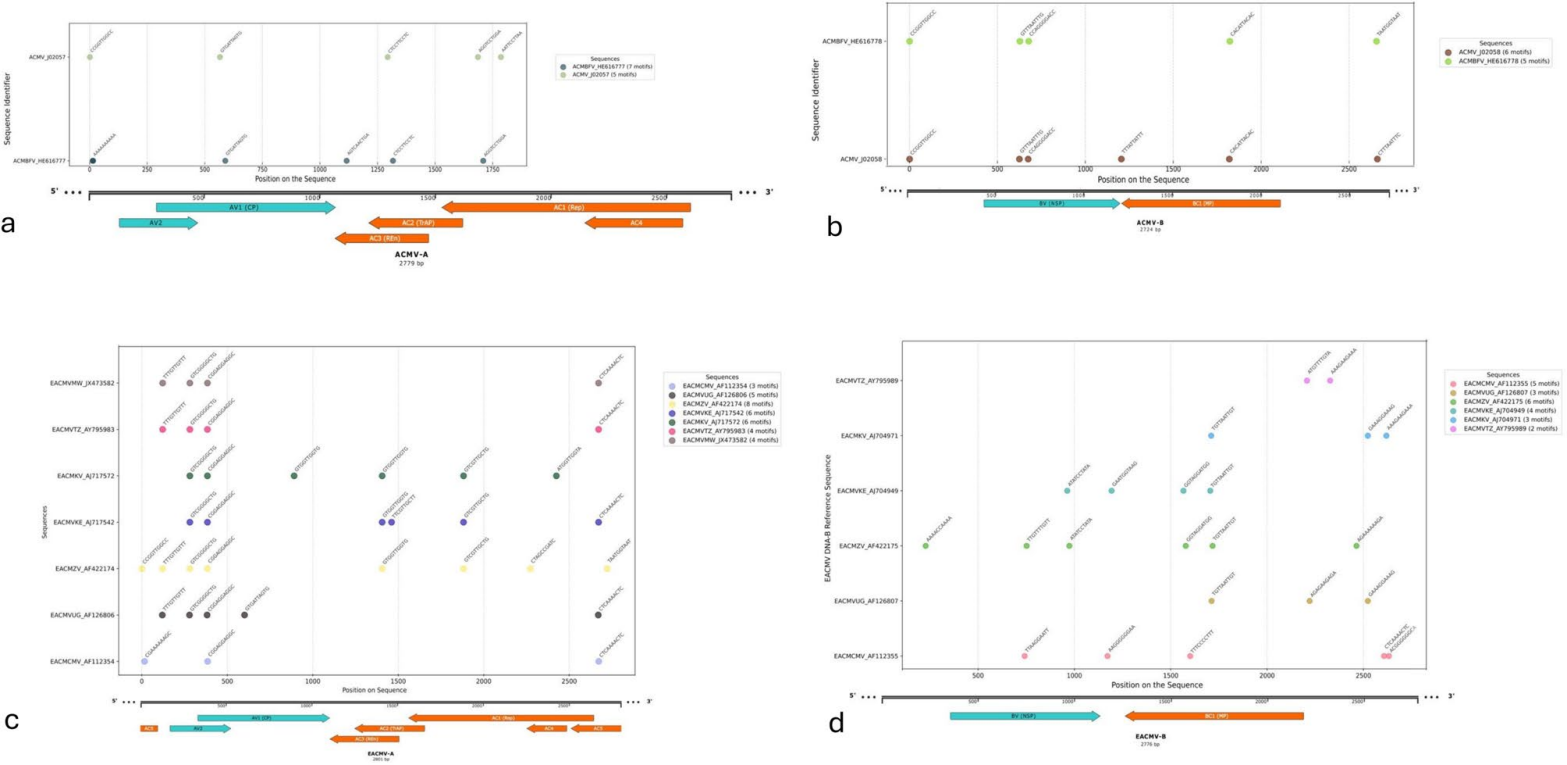
Palindromic motifs and genomic features of cassava mosaic begomoviruses. Visualization of palindromic motifs mapped onto the genomic organization of cassava mosaic begomoviruses. Panels (a), (b), (c), and (d) represent different viral isolates or strains (a: ACMV-A, b: ACMV-B, c: EACMV-A, d: EACMV-B). The motifs, annotated on the plots, are aligned to their respective genomic positions across different sequences. The schematic below each panel highlights the key genomic elements, including coding regions for replication-associated protein (Rep), coat protein (CP), movement protein (MP), and other functional domains. The distribution of motifs provides insight into conserved and variable regions critical for viral diagnostics and molecular studies.

Custom Python scripts identified conserved palindromic motifs within the ACMV and EACMV genomes. In ACMV DNA-A, 12 motifs, including GTGATTAGTG and CTCCTTCCTC, were mapped within the AV1 and AC3 regions, highlighting their structural relevance. ACMV DNA-B showed 11 motifs, such as CCGGTTGGCC and GTTTAATTTG, overlapping the BV1 and BC1 regions (Fig. 3). EACMV DNA-A and DNA-B analyses revealed 36 and several motifs, respectively, distributed across coding regions. Motifs such as GTCGGGGCTG and TGTTAATTGT highlighted conserved elements associated with DNA stability and regulatory functions (Fig. 3).

Additional computational analyses revealed clustering of motifs within regulatory and replication-associated regions, suggesting their involvement in essential viral processes. The identification of recurrent motifs highlights conserved structural features that could serve as diagnostic targets or therapeutic intervention points. These motifs also provide insights into viral genome organisation and replication mechanisms.

Comprehensive sequence analyses combining the identification of conserved genomic regions with palindromic motif analysis provided solid in silico validation of the designed primers. By carefully mapping conserved sites across the viral genomes, we ensured that the primers would target stable regions that are less susceptible to sequence variability, thereby maximising their diagnostic relevance. At the same time, assessing palindromic motifs enabled us to minimise the risk of secondary structures (hairpins) that could impair primer binding efficiency. This approach yielded a reliable panel of primers that cover multiple genomic regions and offer broad applicability across viral strains, as well as improved robustness for downstream diagnostic assays.

### Validation of newly designed primers for the detection of ACMV and EACMV

Validation of the newly designed primers using PCR on ‘recalcitrant’ samples demonstrated their high efficiency in detecting cassava mosaic begomoviruses (CMBs) in symptomatic samples. Consistent amplification across a wide range of isolates confirmed the specificity and robustness of the primer sets.

### ACMV DNA-A and DNA-B detection

Using the new primer pairs, ACMV DNA-A was detected in 42 samples, showing high sequence similarity to known isolates. Twenty-one of these samples matched ACMV-[Ghana:FM1A:13] (MG250156), while the remainder clustered with isolates from Nigeria, Burkina Faso, Benin and the Central African Republic, exhibiting nucleotide identities ranging from 95.6% to 99.7%. Similarly, ACMV DNA-B was identified in 47 samples, showing 92.1–97.7% identity with isolates from Ghana, Nigeria and the Central African Republic. These findings confirm the broad distribution and genetic variability of ACMV across West Africa, highlighting its capacity for rapid evolution and potential to adapt to diverse agro-ecological contexts.

### EACMV DNA-A and DNA-B detection

Although less frequent, EACMV was also successfully detected. DNA-A was identified in one sample, showing 97.5–98% similarity to isolates from Madagascar, and DNA-B was detected in two samples, showing high similarity to isolates from Cameroon and Madagascar. Despite its low prevalence, these results confirm the presence of EACMV in Nigeria and emphasise its potential role in cassava mosaic disease (CMD) outbreaks in the region.

### Recombination signals revealed

A total of 54 recombination events across the DNA-A and DNA-B segments met the a priori validation rule (≥ 3 methods): 47 in DNA-A and seven in DNA-B (Supplementary Table S1: Harmonised validated events, DNA-A + DNA-B). For each event, we report the recombinant sequence, its putative major and minor parents, and the estimated breakpoint interval. Most validated events were supported by multiple non-redundant tests (including combinations of RDP, GENECONV and BootScan, as well as at least one of MaxChi, Chimaera, SiScan and 3Seq), which is consistent with robust topological incongruence rather than method-specific bias (Supplementary Table S1: Per-method support summary). The combined histogram of stary + end positions for DNA-A and DNA-B indicates non-uniform distributions along the alignment (Supplementary Figure S1: Combined breakpoint distribution). This visualisation allows for a qualitative comparison of breakpoint density between segments without presupposing a specific mechanistic model. GARD efficiently identified candidate partitioning on centroid sets. For the largest full alignments, AICc occasionally limited formal comparison of multi-partition models due to high n relative to the number of aligned informative sites, which justifies our two-step strategy (centroids for discovery and RDP4 for stringent validation). Where full re-estimation was permitted by AICc, the results were consistent with the events validated by RDP4.

NeighbourNet networks provided clear evidence of phylogenetic conflict within both the DNA-A and DNA-B datasets, which is consistent with pervasive recombination. For the DNA-A dataset (Supplementary Figure S2), the network displayed extensive reticulation between the ACMV and EACMV clusters. Several parallelogram-like structures were observed around the replication-associated protein (AC1) and the intergenic region. These structures mirrored the breakpoints identified by GARD and supported by 3SEQ. The presence of intermediate clusters between ACMV and EACMV suggests that stable recombinant genotypes are circulating. In the case of DNA-B, the network exhibited conflicting topologies to a lesser extent than DNA-A. Distinct reticulations linked ACMV and SACMV isolates, reflecting ancient recombination events affecting the movement protein (BC1) and nuclear shuttle protein (BV1) genes (Supplementary Figure S1 and S3). These findings are consistent with PhiPack results, which produced significant signals across the PHI, NSS and MaxChi tests. Overall, NeighborNet analyses confirm that the genetic diversity of cassava mosaic begomoviruses in Africa is strongly influenced by inter- and intra-species recombination. These events explain the discrepancies observed in tree-based phylogenies and provide a framework for understanding the evolutionary plasticity of these viruses.

## Discussion

The results of this study confirm the effectiveness of Oxford Nanopore Technology (ONT) sequencing in overcoming the limitations of conventional diagnostic methods for the detection of cassava mosaic begomoviruses (CMBs). The acquisition of high-quality reads enabled the precise identification and characterisation of African cassava mosaic virus (ACMV) and East African cassava mosaic virus (EACMV) strains in symptomatic samples that previously tested negative by conventional PCR methods. These results underscore the increased sensitivity of ONT, as previously highlighted by Boykin et al.^7–10^. The detection of mixed viral populations and potential recombination events emphasizes the genomic complexity of CMB infections and supports previous studies suggesting that recombination is a key driver of viral evolution and adaptation^11,12^⁠.

Furthermore, the design of new primers targeting conserved genomic regions, including AV1, AC2, BV1, and BC1 (Fig. 2), significantly improved detection rates, by as high as 95% depending on the primer pairs and sample sets (Table 3). Identification and exclusion of palindromic motifs that could form secondary structures optimised primer specificity and sensitivity^13,14^⁠. Computational analyses, including custom Python scripts, and software such as Clustal Omega, Primer-BLAST, and Geneious Prime 2022, allowed precise mapping of binding sites and prediction of conserved motifs potentially involved in viral replication and regulation. Validation by Sanger sequencing further confirmed the accuracy and reliability of these newly designed primers, reinforcing the robustness of this approach for molecular diagnostics.

**Table 3:** Detection rates of cassava mosaic begomoviruses (CMBs) in ‘recalcitrant samples across four West African countries.

Country	Viruses detected in ‘recalcitrant’ samples			
	ACMV	EACMV	ACMV + EACMV	Negative
Nigeria (50 samples)	58%	2%	38%	2%
Côte d’Ivoire (80 samples)	12.5%	6.25%	66.25%	15%
Ghana (110 samples)	0%	0.9%	98.2%	0.9%
Burkina Faso (36 samples)	89%	0	0	11%

This table summarises the detection rates of ACMV and EACMV in symptomatic cassava samples that first tested negative with conventional PCR and were subsequently analysed using Oxford Nanopore Technology (ONT).

This study is consistent with previous research highlighting the genetic diversity and high recombination rates of cassava mosaic begomoviruses in sub-Saharan Africa^2,3^⁠. The sequences obtained not only confirmed the presence of highly diverse viral populations but also highlighted inter-regional genetic flow, reflecting the potential role of trade and human-mediated propagation in viral spread. These findings highlight the need for region-specific diagnostic, surveillance and certification programmes to reduce the spread of CMD-associated viruses. It complements epidemiological surveys conducted under the WAVE programme, such as that of Soro et al.^5^⁠, which highlighted the inadequacy of older diagnostic tools for certain symptomatic samples. The ability of ONT to generate real-time data, even under field conditions^7^⁠, represents a significant advancement over traditional molecular techniques, which are often limited by sensitivity and specificity^5^⁠.

Compared to other next-generation sequencing technologies, such as Illumina, ONT’s portability and rapid processing capabilities make it particularly suitable for resource-limited settings^7^⁠. Furthermore, the identification of recombinant variants that eluded detection by conventional PCR mirrors findings by Mehetre et al.^15^⁠ and underscores the need for more robust tools to track highly dynamic viral populations^12^⁠. This study extends the literature by integrating motif analysis and secondary structure predictions, providing novel insights into genome stability and replication mechanisms (Table 4).

**Table 4:** Previously published primer pairs for the detection of cassava mosaic begomoviruses (CMBs).

Primer name	Primer sequence	Size (bp)	Target region	References
JSP001F	ATGTCGAAGCGACCAGGAGAT	783	AV2 (CP)	Pita et al.^4^
JSP002R	TGTTTATTAATTGCCAATACT			
ACMVBF	TCGGGAGTGATACATGCGAAGGC	628	AV1/BC1	Matic et al.^16^
ACMVBR	GGCTACACCAGCTACCTGAAGCT			
JSP001F	ATGTCGAAGCGACCAGGAGAT	780	AV2 (CP)	Pita et al.^4^
JSP003R	CCTTTATTAATTTGTCACTGC			
CMBRepF	CRTCAATGACGTTGTACC A	650	AC1	Alabi et al.^17^
EACMVRepR	GGTTTG CAGAGAACTACATC			
VNF031F	GGATACAGATAGGGTTCCCAC	~ 560	AC2/AC3	Fondong et al.^18^
VNF032R	GACGAGGACAAGAATTCCAAT			
EAB555F	TACATCGGCCTTTGAGTCGCATGG	544–560	BC1/CR	Ndunguru et al^19^.
EAB555R	CTTATTAACGCCTATATAAACACC			

These primers have been widely used in earlier studies for virus identification and epidemiological studies, providing a foundation for comparative analysis with newly designed primers in this study.

The findings of this study have important implications for early diagnosis and epidemiological surveillance of begomovirus-induced diseases, particularly in Central and West Africa. The ability to rapidly and accurately detect viral diversity in endemic regions can inform breeding programmes, cultural practices, and targeted phytosanitary measures^1^⁠. The newly designed primers demonstrated higher diagnostic resolution, making them suitable for routine protocols to identify emerging variants early and reducing the risk of propagation through infected cuttings.

Recombination has long been recognised as a major driver of begomovirus evolution, and our analyses provide direct evidence that the DNA-A and DNA-B components of cassava mosaic begomoviruses in West Africa undergo frequent genetic exchange. These events likely contribute to the emergence of novel strains with an altered host range or pathogenicity, thereby complicating disease management strategies^11,20^. Notably, recombination can undermine the durability of host resistance and diminish the effectiveness of existing diagnostic primers, as recombinant genomes frequently contain mosaic regions that conventional assays fail to detect^21^. These findings highlight the importance of integrated surveillance strategies combining high-resolution sequencing and adaptable diagnostic tools to anticipate and mitigate the impact of recombinant lineages on cassava production^22^. Combining a phylogeny-aware screen (GARD), multi-test corroboration (RDP4 ≥ 3 methods) and NeighborNet networks demonstrates that recombination is present in the dataset, predominantly in DNA-A, with fewer but credible events in DNA-B. These results affect the interpretation of subsequent phylogenetic analyses: trees spanning recombinant regions should be treated with caution, and conclusions at the clade level should be based on analyses that are robust to the removal of recombinant tracts. In addition, insights into recombination mechanisms and molecular determinants of virulence provide a basis for the development of improved management strategies, including resistant varieties^23^⁠. The integration of ONT into broader surveillance programmes could also enable the prediction and containment of large-scale outbreaks in sub-Saharan Africa.

Despite its benefits, this study has certain limitations. The initial cost of ONT and need for bioinformatics expertise remain a barrier to widespread adoption in all research stations^24^⁠. In addition, the large datasets generated, and variable sequencing quality require robust bioinformatics pipelines to filter out low-quality reads and validate assemblies^25^. Expanding sampling efforts to include more geographic regions and seasonal variations would provide a more comprehensive understanding of the spatial and temporal dynamics of CMBs.

Further optimisation of multiplex PCR assays could complement ONT-based diagnostics to address the approximately 10% of symptomatic samples that remain undetected. Functional studies, such as site-directed mutagenesis of palindromic motifs, would help elucidate their roles in viral replication and pathogenesis. These approaches would refine diagnostic capabilities and improve integrated disease management strategies for cassava mosaic disease, ultimately ensuring food security^2,7^⁠.

This study demonstrates the transformative potential of ONT-based diagnostics for cassava mosaic disease. By integrating molecular and computational approaches, it establishes a framework for accurate viral detection, genomic characterisation, and epidemiological surveillance. The results highlight the importance of continued research to optimise diagnostic tools and explore antiviral interventions targeting key genomic elements. Future work should expand sampling, validate motif functions, and integrate ONT into regional disease surveillance programmes to effectively mitigate the impact of CMD.

## Methodology

### Sample collection and DNA extraction

Cassava leaf samples exhibiting symptoms of cassava mosaic disease (CMD) were collected during the 2020 WAVE epidemiological survey in South-West and North-Central Nigeria. A total of 50 symptomatic leaf samples, which tested negative using conventional PCR with primers (Table 4), were selected for further analysis. Total DNA was extracted from each sample using the modified CTAB protocol^26^. DNA concentration and purity were measured using a NanoDrop 2000 spectrophotometer (Thermo Fisher Scientific), and samples were normalized to 100 ng/μL for downstream processing, including Rolling Circle Amplification (RCA)^27^⁠.

### Library preparation and nanopore sequencing

In 2022, we initially prepared sequencing libraries using the 1D Native Genomic DNA barcoding protocol (EXP-NBD104 and SQK-LSK109) from ONT. Briefly, 1 µg of total DNA was end-prepared by mixing with Ultra II End-Prep Reaction Buffer (7 μL), Ultra II End-Prep Enzyme Mix (3 μL; New England Biolabs, UK) and nuclease-free water (5 μL). The reaction was then incubated at 20 °C for 5 min, followed by 65 °C for a further 5 min. The DNA was then purified using AMPure XP beads (Beckman Coulter, USA), washed with 70% ethanol and eluted on a magnetic rack. DNA librairy quantification was performed using an Invitrogen Qubit^TM^ 4 Fluorometer (Thermo Fisher Scientific, France) and the Qubit^TM^ dsDNA HS Assay Kit (Thermo Fisher Scientific, Cat. No. Q32854). Barcoding was performed by ligating barcode adapters using Blunt/TA Ligase Master Mix, followed by purification with AMPure XP beads. PCR barcoding was then carried out using LongAmp Taq 2 × Master Mix and barcodes (BC01–BC12) under the following thermal cycling conditions: 95 °C for 3 min; 18 cycles of 95 °C for 15 s, 62 °C for 15 s and 65 °C for 7 min; and a final extension at 65 °C for 15 min. The barcoded products were then pooled in equimolar amounts, cleaned up again, and sequenced on a MinION Mk1C device (MinKNOW v21.11.7) using FLO-MIN106 (R9.4.1) flowcells. Despite successful library construction and sequencing, the overall yield and read quality were suboptimal.

To improve sequencing performance, we adopted the Rolling Circle Amplification (RCA)-MinION approach in combination with the Native Barcoding 96 Kit V14 (SQK-NBD114.96). RCA was first used to amplify circularised DNA templates, providing a high yield of uniform fragments. Barcoding and library preparation were then performed in accordance with the SQK-NBD114.96 protocol, enabling high-throughput multiplexing. The barcoded products were then purified using AMPure XP beads, quantified using Qubit, normalised and pooled prior to sequencing. Sequencing was carried out on a MinION Mk1C device with FLO-MIN114 (R10.4) flowcells using MinKNOW v24.02/16 software. The flowcells were primed with Flush Buffer and Flush Tether (ONT, EXP-FLP001) and 12 µL of the final library was mixed with Sequencing Buffer and Library Loading Beads before being loaded onto the SpotON port. Runs were conducted for 48 h.

Compared to the earlier SQK-LSK109/EXP-NBD104 workflow, this updated strategy markedly improved sequencing sensitivity, throughput, and barcode representation.

### Bioinformatic processing of sequencing data

For libraries prepared using the DNA barcoding protocol (EXP-NBD104 and SQK-LSK109), the raw sequence data (Fast5 files) were basecalled in super-accuracy mode using Guppy v6.3.2. After quality filtering and adapter trimming, reads with a minimum Q-score of 7 were retained. The filtered reads were then aligned against the cassava and human DNA reference genomes using Minimap2 v2.24 to remove contaminant sequences. The remaining reads were then processed for taxonomic assignment using KronaTools and DIAMOND, which enabled metagenomic profiling (Fig. 1).

For libraries generated using the Rapid Barcoding 96 Kit V14 (SQK-RBK114.96), raw POD5 data were basecalled with Dorado in super-accuracy mode using the model dna_r10.4.1_e8.2_400bps_sup-v5.0.0. Raw ONT reads were then quality-checked and adapter-trimmed. To remove potential contaminants, reads were aligned against host (Manihot esculenta, cassava) and human references genome using Minimap2 (v2.30); reads mapping to host or human were discarded. The remaining non-host/non-human reads were assembled de novo with Flye (ONT preset; v2.9.5). If Flye did not yield a valid assembly, contigs were generated with Raven (v1.8.3). Resulting assemblies were polished iteratively with Racon (v1.5.0) and Medaka (v2.1.1) to improve consensus accuracy. Contigs were further filtered by length, retaining only viral-size sequences. For circular viral genomes, terminal duplications were trimmed and concatemerized contigs resolved when detected (Fig. 1).

We compiled full-length cassava-infecting begomovirus genomes for each segment (DNA-A and DNA-B) retrieved from Genbank, removing exact duplicates and malformed entries and retaining sequences spanning the canonical coding regions. We generated multiple sequence alignments with MAFFT (*–auto*), where indicated, to confirm alignment stability. To mitigate the expected recombination artefacts in cassava begomoviruses, we screened the alignments using HyPhy GARD. When breakpoints were identified, we masked the implicated segments before inferring the tree. The alignments were then trimmed using ClipKIT (in kpic mode) to retain phylogenetically informative sites while minimising gaps and saturated positions. For gene-wise analyses (AV1/CP, AC1/Rep, BV1/NSP and BC1/MP), the sequences were codon-aligned and handled separately to avoid the inter-segment incongruence that is inherent in bipartite genomes. Maximum-likelihood (ML) trees were inferred using IQ-TREE 2 with ModelFinder to select the best-fit model (MFP). Branch support was assessed using ultrafast bootstrap (UFBoot, 1,000 replicates with -bnni) and SH-aLRT (1,000 replicates). Unless stated otherwise, nodes with UFBoot ≥ 95% and SH-aLRT ≥ 80% were considered strongly supported. Consensus and annotated trees were produced from the ML runs (consensus was produced when bootstrapping was employed), rooted using the midpoint or an appropriate outgroup (ACMV/EACMV references were used when available) and visualised using ggtree (R 4.3) to overlay metadata and export publication-quality figures (PNG/PDF). All steps were executed in a reproducible Conda environment (key tools: seqkit, MAFFT/Muscle5, ClipKit, Hyphy 2.5, IQTree2 and R + Ggtree), with fixed random seeds and command-line parameters provided to ensure full repeatability.

### Primer design and validation

Multiple sequence alignments of cassava mosaic begomoviruses (ACMV and EACMV) were performed using Clustal Omega^28^⁠ to identify conserved regions, including the intergenic regions (IR) and genes encoding replication-associated protein (AC1), replication enhancer protein (AC2), transcriptional activator protein (AC3), AC4 involved in pathogenicity, and coat protein (AV1) for DNA-A. For DNA-B, primers targeted the movement protein (BC1) and nuclear shuttle protein (BV1). Complete genome sequences of ACMV and EACMV (DNA-A and DNA-B) were retrieved from GenBank and used as the initial dataset for the analysis. Custom Python scripts were then used to detect palindromic motifs, which could affect secondary structures and primer efficiency. Primer pairs were designed using Geneious Prime 2022 and Primer3^29^⁠ and their specificity were evaluated with Primer-BLAST^30^⁠.

**Fig. 4:**
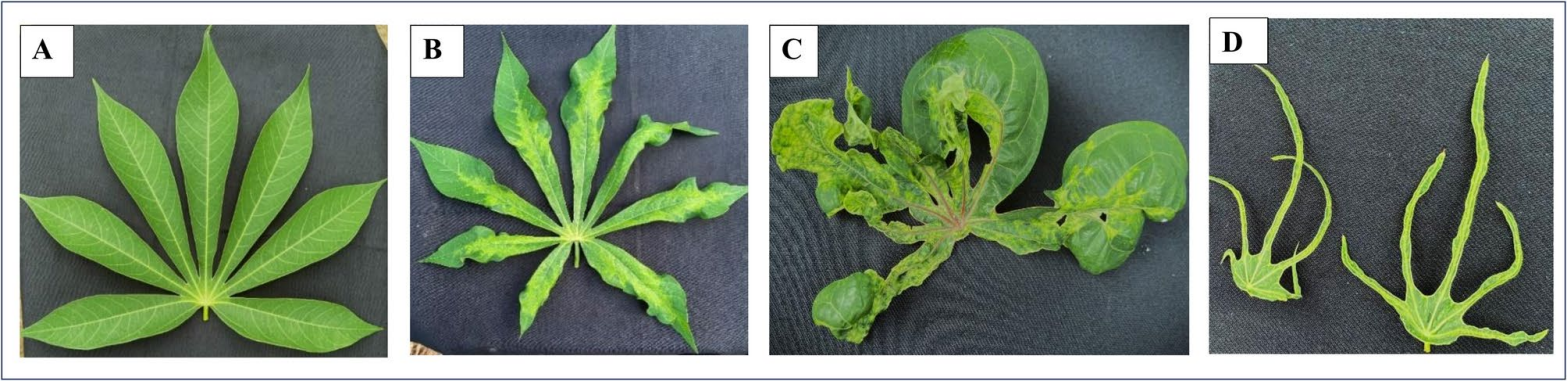
Symptoms of cassava mosaic disease observed in cassava samples. (**A**) healthy leaf; (**B**) severe mosaic and slight deformation of leaves; (**C**) very severe mosaic, deformation and curling of leaves; (**D**) filiform leaves.

**Fig. 5:**
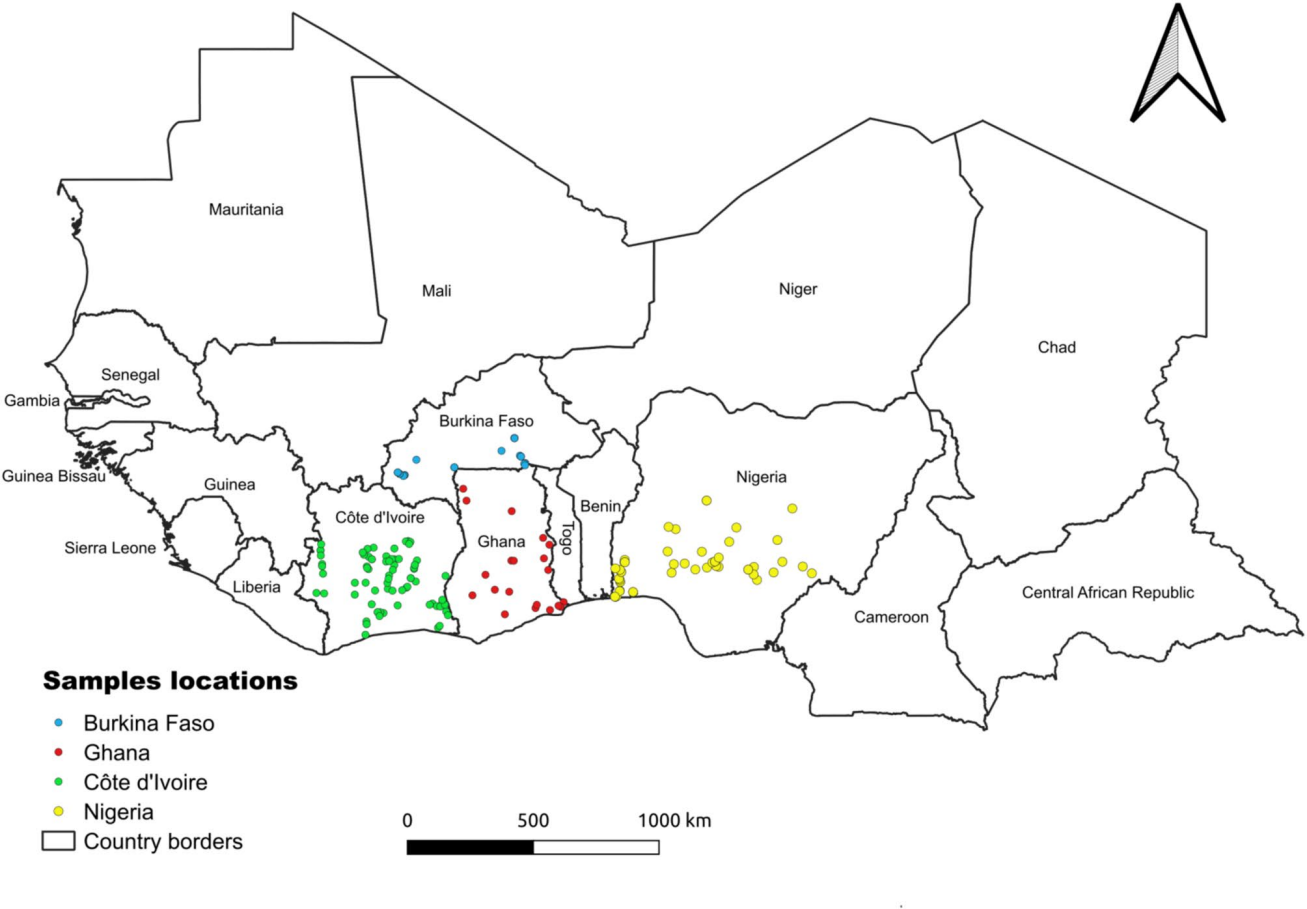
Map showing the locations of the sample collection points in Burkina Faso, Côte d’Ivoire, Ghana and Nigeria.

In silico PCR simulations^13^⁠ confirmed target amplification without off-target binding. Primer sensitivity was validated experimentally on cassava samples from Burkina Faso (36 samples), Côte d’Ivoire (80 samples), Ghana (110 samples), and Nigeria (50 samples) (Fig. 4 for symptoms observed and Fig. 5 for samples locations). PCR reactions were performed in 25 µL volumes containing 1 × GoTaq buffer, 0.625 U GoTaq polymerase, 0.4 μM primers, 0.2 mM dNTPs, and 1 mM MgCl2. Amplification conditions were 94 °C for 4 min, 35 cycles of 94 °C for 1 min, 55 °C for 1 min, and 72 °C for 1 min, followed by a final extension at 72 °C for 10 min.

PCR products were visualized by electrophoresis on 1% agarose gels stained with ethidium bromide and examined under UV light. Representative products were Sanger sequenced (Genewiz) to confirm primer specificity and sequence identity.

### Statistical and computational analysis

Palindromic motifs in reference CMB sequences were analysed using a custom Python script developed to detect inverted repeat sequences that may form secondary structures impacting primer binding efficiency and specificity. This analysis provided insights into conserved and variable regions across cassava mosaic begomoviruses (CMB) genomes. Forward and reverse primer binding sites were mapped within coding regions, with structural motifs evaluated to predict their influence on primer performance.

For ACMV DNA-A and DNA-B, the Python script identified primer binding sites within the AV1 and AC3 regions, and BV1 and BC1 regions, respectively. It also detected palindromic motifs in ACMV DNA-A and motifs in ACMV DNA-B, including highly conserved sequences. Similar analyses for EACMV DNA-A and DNA-B were performed.

Computational analyses, including motif mapping, secondary structure prediction with Mfold, and simulated PCR using Primer-BLAST, validated primer specificity and efficiency, ensuring their suitability for molecular diagnostics.

### Recombination and network analyses

Data pre-processing: All complete CMB genomes (DNA-A and DNA-B) were obtained and curated in FASTA format. The files were then normalised (end-of-line unification and removal of empty records) and duplicates were collapsed using seqkit v2.1.0 (rmdup -s). Approximately 766 DNA-A and 296 DNA-B sequences were aligned using MAFFT v7.505 with the following parameters: *–adjustdirection, –maxiterate 0, –retree 2, –anysymbol and multi-threaded*. To limit artefacts from poorly aligned regions, alignment columns with a gap-like character fraction (‘-’, ‘.’, ‘?’, or space) of > 50% were masked. Ambiguous IUPAC bases (R, Y, S, W, M, K and N) were not treated as gaps during this process. To meet CD-HIT-EST input requirements, a de-aligned version was generated for clustering (gaps removed; non-ACGT mapped to N). Redundancy reduction was performed for recombination scanning. To accelerate the search for breakpoints while preserving the phylogenetic signal, non-redundant representative sets (‘centroids’) were created using CD-HIT-EST v4.8.1 with a 99% identity threshold (-c 0.99 -n 10). The centroids were realigned using the same MAFFT options as above. Primary breakpoint screening (GARD, HyPhy). GARD (HyPhy v2.5.70) was used to screen the alignments for phylogenetic incongruence among the putative partitions. For the centroid sets, the models were GTR, with among-site rate heterogeneity modelled using a discrete Gamma distribution with four categories. The *–max-breakpoints* option was set to between 8 and 12, depending on the length of the alignment, and the *–mode option* was set to Faster for the genetic algorithm search. When the corrected Akaike information criterion (AICc) permitted comparison of ≥ 2 partitions given the number of sequences and sites, we repeated the estimation process on the full masked alignments using a lighter model (HKY85, two Gamma classes, *–mode Normal* and *–max-breakpoints* 8) to avoid over-parameterisation under high n/moderate L conditions. For large alignments that occasionally raised floating-point tolerance warnings, HyPhy was executed with *ENV* = *TOLERATE_NUMERICAL_ERRORS* = *1*, which is a recommended safeguard for numerical stability on big datasets.

To validate the GARD-suggested signals using orthogonal tests, we analysed the same alignments in RDP4 (the most recent stable version). We exported the ‘Breakpoint positions/Detection methods’ table and parsed it to extract the recombinant, major/minor parents, begin/end positions and method-specific support for each event (RDP, GENECONV, BootScan, MaxChi, Chimaera, SiScan, 3Seq and, when present, PhylPro/LARD). An event was considered validated if it was supported by at least three methods with p < 0.05 (when numeric p-values were provided) or if it had a non-NS indicator in the RDP4 export (standard for this output). The DNA-A and DNA-B tables were then harmonised into a single dataset (Supplemental Table S1) containing the following common fields: *event_id, segment, recombinant, major_parent, minor_parent, begin, end, n_sig_methods and sig_methods*.: We verified the following for Robustness checks:

- (i) Masked alignments retained ample variable sites.
- (ii) Inferred partitions were not < 5% of the alignment length.
- (iii) Events validated by RDP4 were not driven by idiosyncrasies of a single method.
- (iv) Results were qualitatively stable when tightening the validation threshold to ≥ 4 methods (sensitivity analysis; not shown). The distribution of breakpoint positions was summarised using pooled histograms of begin and end sites for each segment.

To complement breakpoint-based approaches such as GARD, 3SEQ and PhiPack, we performed reticulate phylogenetic analyses using the NeighborNet algorithm, as implemented in the phangorn R package. Pairwise distances were computed on the dataset using the raw model, and NeighborNet networks were inferred with a maximum of 400 tips and a per-species cap of 150 sequences to improve interpretability. The resulting networks were visualised using ggtree, with sequence labels coloured according to their taxonomic assignment (ACMV, EACMV or SACMV). R scripts were designed to ensure reproducibility and generate SVG outputs, which were then simplified and standardised using Inkscape.

## Conclusion

The findings presented in this study highlight the effectiveness of ONT sequencing in overcoming the limitations of conventional diagnostic methods for the detection of begomoviruses responsible for cassava mosaic disease (CMD). The high-resolution and highly sensitive data generated enabled the rapid and accurate identification of viral diversity, including recombinant variants and mixed infections that had previously eluded detection by conventional PCR. Furthermore, the design of new primers targeting conserved genomic regions—while taking into account palindromic motifs that could affect amplification—significantly improved detection rates.

This integrated approach, combining high-throughput sequencing and computational analysis, provides a solid foundation for strengthening epidemiological surveillance and the management of cassava viral diseases. The wider adoption of portable sequencing tools such as ONT, coupled with tailored bioinformatics expertise, will contribute to early detection, the development of resistant cassava varieties, and the improvement of CMD control strategies. Ultimately, these advances offer new prospects for ensuring food security and sustaining cassava production in sub-Saharan Africa.

## Supplementary Information

Supplementary Information 1.

Supplementary Information 2:Combined distribution of recombination breakpoints and genomic open reading frame (ORF) map. Overlaid histograms of validated breakpoint positions (start and end) for DNA-A (blue) and DNA-B (orange), plotted against genomic coordinates (nt). Identical binning is used for both segments. The lower panel shows a schematic of begomovirus open reading frames to provide context for the breakpoint density: DNA-A (AV2, AV1/CP, AC1/Rep, AC2/TrAP, AC3/REn and AC4) and DNA-B (BV/NSP and BC1/MP). Only events that meet the ≥3-method RDP4 criterion are plotted, enabling a visual comparison of putative ‘hotspots’ between the two segments without assuming a specific mechanistic model.

Supplementary Information 3:Phylogenetic network of begomovirus DNA-A sequences. Caption: The network was derived from the masked DNA-A alignment, with nodes coloured by taxon: ACMV (blue), EACMV (red) and SACMV (green). Selected accessions are labelled for orientation. Prominent reticulations indicate topological incongruence, which is consistent with recombination, and correspond to events that are validated in Supplementary Table S1.

Supplementary Information 4:Phylogenetic network of begomovirus DNA-B sequences. Caption: This network is based on the masked DNA-B alignment. Node colours follow the same legend (ACMV: blue; EACMV: red; SACMV: green). Reticulate structures indicate incongruences that are compatible with recombination and are generally less frequent than in DNA-A. This is consistent with the smaller number of validated events reported in Supplementary Table S1.

Supplementary Information 5:Harmonised list of validated recombination events (DNA-A + DNA-B).Unified list of validated recombination events across both segments. An event is considered validated when it is supported by at least three RDP4 methods (RDP, GENECONV, BootScan, MaxChi, Chimaera, SiScan and 3Seq, and PhylPro/LARD if present) with a p-value of less than 0.05 if numerical p-values are available, or by a non-‘NS’ indicator in the RDP4 export. Fields: event_id; segment (DNA-A/DNA-B); recombinant; major_parent; minor_parent; begin; end (coordinates in the masked alignment); n_sig_methods (number of supporting methods); and sig_methods (method names). Missing values are recorded as NA. This table forms the basis of the method-wise summaries and the breakpoint distribution figure.

## Data Availability

All data supporting the findings of this study are openly available. Raw Oxford Nanopore Technologies (ONT) reads have been deposited in the NCBI Sequence Read Archive under the BioProject accession number PRJNA1312059. The assembled viral genomes are available in GenBank under the accession numbers PX289778–PX289794. Multiple-sequence alignments, HyPhy GARD outputs, RDP4 exports, the harmonised table of validated recombination events and figure source data have been deposited in Zenodo (10.5281/zenodo.17220156). The analysis code and reproducible workflows are available under an open-source licence at [https://github.com/etibiri/Recomb/tree/feat/pipeline-bootstrap], as well as in Supplemental Data S1.
