# Supplementary material for: Improving the diagnosis of cassava mosaic begomoviruses using Oxford Nanopore Technology sequencing
Source: Sci Rep. 2025 Nov 21;15:41432. doi: 10.1038/s41598-025-25233-8 (PMC12638911; doi:10.1038/s41598-025-25233-8)
Supplement: Supplementary file 1 — Supplementary Information 1. [file 41598_2025_25233_MOESM1_ESM.docx]

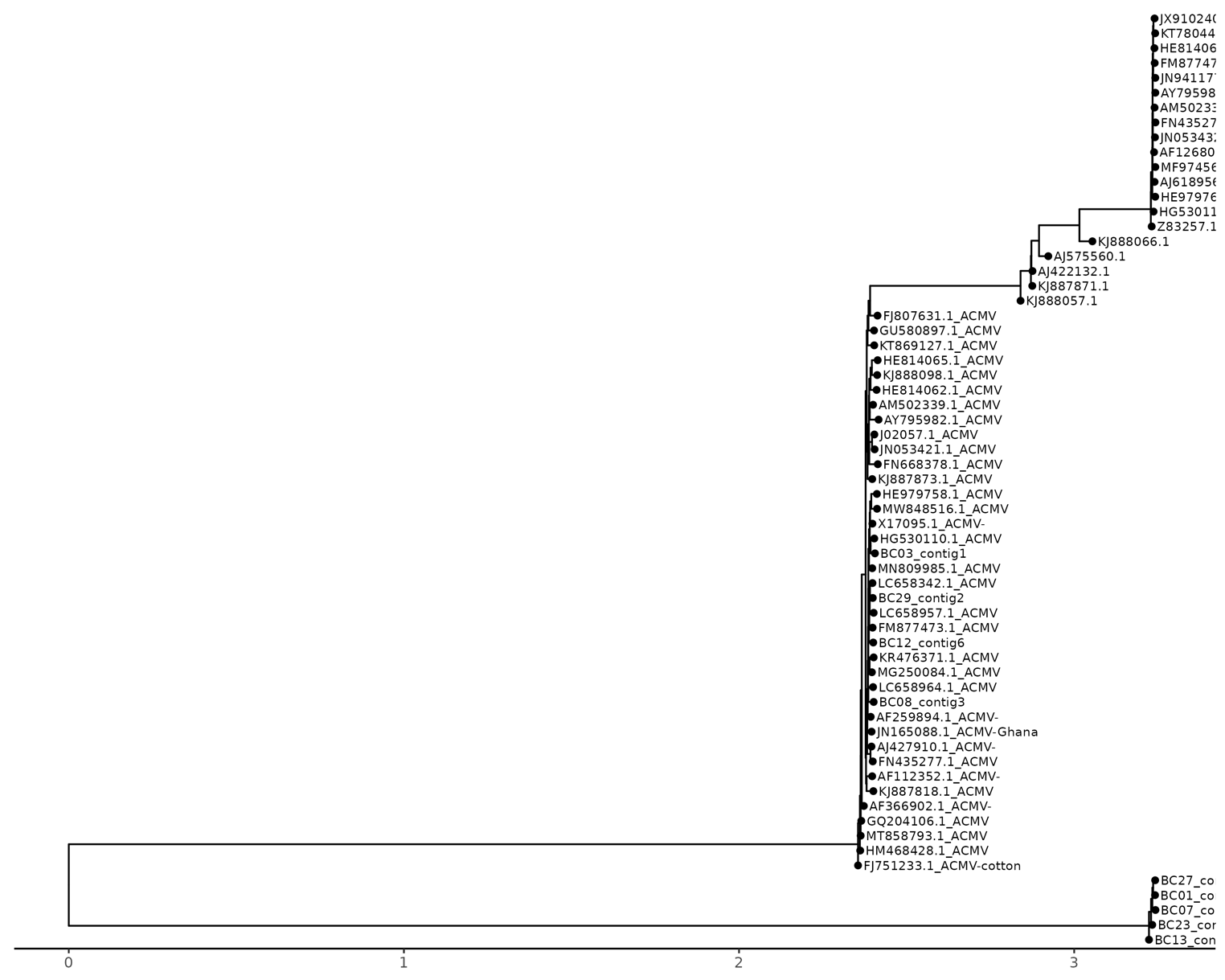


Diversity and phylogenetic structure of cassava begomoviruses: ML trees for DNA-A (63×4 096 pb; modèle GTR+F+I+R4)


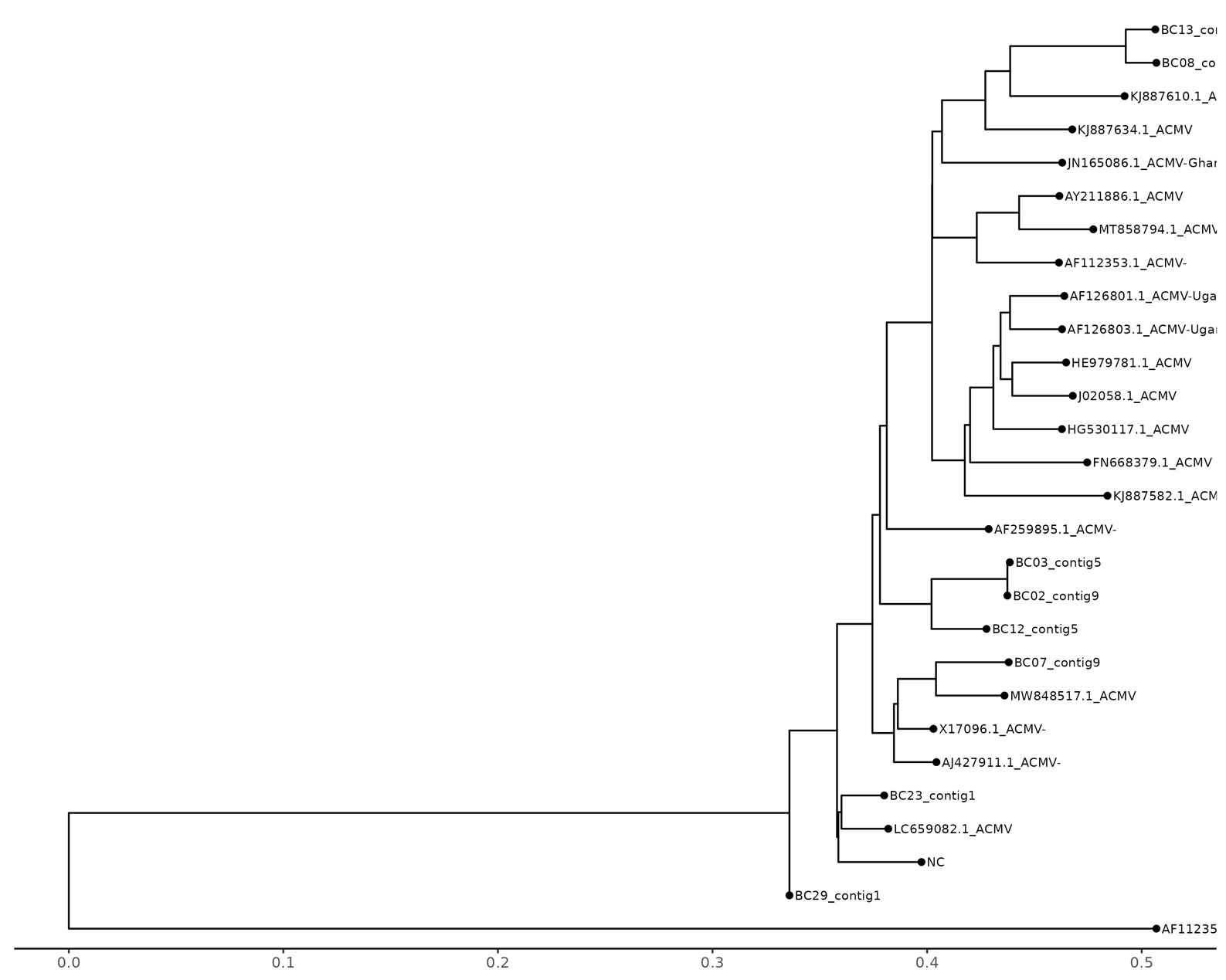


Diversity and phylogenetic structure of cassava begomoviruses: ML trees for DNA-B (28×2 934 pb; modèle GTR+F+I+G4)

We compiled full-length cassava-infecting begomovirus genomes for each segment (DNA-A and DNA-B), removing exact duplicates and malformed entries and retaining sequences spanning the canonical coding regions. Headers were standardised to unique IDs and linked to sample metadata (virus, segment, country and year). We generated multiple sequence alignments with MAFFT (--auto) or MUSCLE v5, where indicated, to confirm alignment stability. To mitigate the expected recombination artefacts in cassava begomoviruses, we screened the alignments using HyPhy GARD. When breakpoints were identified, we masked the implicated segments before inferring the tree. The alignments were then trimmed using ClipKIT (in kpic mode) to retain phylogenetically informative sites while minimising gaps and saturated positions. For gene-wise analyses (e.g. AV1/CP, AC1/Rep, BV1/NSP and BC1/MP), the sequences were codon-aligned and handled separately to avoid the inter-segment incongruence that is inherent in bipartite genomes.

Maximum-likelihood (ML) trees were inferred using IQ-TREE 2 with ModelFinder to select the best-fit model (MFP). Branch support was assessed using ultrafast bootstrap (UFBoot, 1,000 replicates with -bnni) and SH-aLRT (1,000 replicates). Unless stated otherwise, nodes with UFBoot ≥ 95% and SH-aLRT ≥ 80% were considered strongly supported. Consensus and annotated trees were produced from the ML runs (consensus was produced when bootstrapping was employed), rooted using the midpoint or an appropriate outgroup (ACMV/EACMV references were used when available) and visualised using ggtree (R 4.3) to overlay metadata and export publication-quality figures (PNG/PDF). All steps were executed in a reproducible Conda environment (key tools: seqkit, MAFFT/Muscle5, ClipKit, Hyphy 2.5, IQTree2 and R + Ggtree), with fixed random seeds and command-line parameters provided to ensure full repeatability.

**ACMV/EACMV - DNA-A:**

Based on 63 full-length DNA-A genomes (4,096 bp each), maximum-likelihood phylogenetic inference selected GTR+F+I+R4 (free-rate) as the best-fit model (ModelFinder), yielding a tree log-likelihood of −25,187.78 ± 489.20. The consensus topology contains 60 internal nodes, 8 of which (13.3%) meet the UFBoot ≥ 95% strong-support criterion. SH-aLRT values were not attached to internal node labels in the exported consensus; therefore, 0% of nodes reach SH-aLRT ≥ 80% in this file, and support summaries rely on UFBoot. The selection of a free-rate model with a proportion of invariable sites suggests significant heterogeneity in the rates of evolution of different sites across DNA-A (e.g. AC1/AV1), which is consistent with the presence of both conserved and rapidly evolving regions, as well as with the known evolutionary diversification within the ACMV/EACMV lineages.

**ACMV - DNA-B**

For DNA-B (28 full-length sequences of 2,934 bp each), ModelFinder selected the GTR+F+I+G4 model, with a tree log-likelihood of −11,428.77 ± 312.85. The consensus tree contains 25 internal nodes, 7 of which (28%) have UFBoot ≥ 95%. Likewise, SH-aLRT values were not encoded in the consensus labels (thus 0% ≥ 80% in this export). The gamma-rate model with a non-zero invariant fraction reflects a combination of highly conserved positions and variable sites in BV1/BC1. Compared with DNA-A, DNA-B shows a more diffuse phylogenetic signal, which is consistent with the bipartite genome architecture and the potential impact of recombination. Recombination-aware analyses (e.g. GARD) and gene-wise trees (BV1 vs BC1) can further refine poorly supported splits.
